# One- and two-locus models for mapping rheumatoid arthritis-susceptibility genes on chromosome 6

**DOI:** 10.1186/1753-6561-1-s1-s103

**Published:** 2007-12-18

**Authors:** Daniel J Schaid, Wan-Yu Lin

**Affiliations:** 1 Division of Biostatistics, Harwick 7, Mayo Clinic, 200 First Street Southwest, Rochester, Minnesota 55905, USA; 2 Institute of Epidemiology, National Taiwan University, Floor 5, No. 17, Shiujou Road, Zhongzheng District, Taipei 100, Taiwan

## Abstract

To evaluate whether there is evidence for two rheumatoid arthritis (RA) susceptibility genes on chromosome 6, we applied new robust methods for two-locus multipoint identical-by-descent mapping to the rheumatoid arthritis data of the Genetic Analysis Workshop 15. The software GEEARP was used to estimate the locations and the corresponding genetic effects for one locus or two linked loci in a region on chromosome 6, on the basis of affected relative pairs. These methods were applied to the data sets from the North American Rheumatoid Arthritis Consortium, Canada, France, and the first screen of United Kingdom. From the resultant 95% confidence intervals given by a robust variance estimator, a linked region, other than the well-known HLA region, was at 54.7–69.6 cM, providing evidence for a second rheumatoid arthritis susceptibility locus on chromosome 6.

## Background

DRB1 alleles located in the HLA region of chromosome 6 affect susceptibility to rheumatoid arthritis (RA), and linkage to HLA has been confirmed [1-4]. Because of multiple linkage peaks on chromosome 6, there may be other genes with minor effects located on this chromosome. However, the strong linkage in the HLA region on 6p21 has made the existence of other genes near this region difficult to resolve. Liang et al. [5] proposed a method of multipoint identical-by-descent (IBD) mapping using affected sib pairs (ASPs) based on generalized estimating equations (GEE). They constructed the mean of IBD scores for ASPs to be a function of gene location and the corresponding genetic effect. Schaid et al. [6] generalized the function to five kinds of ARPs, together with unconstrained and constrained models. These methods give estimates of location and genetic effect, as well as their confidence intervals (CIs), when there is one susceptibility gene in a region. However, when there are two genes in a region, the mean function is misspecified, leading to biased estimates if one applies the one-locus model. Biernacka et al. [7] proposed a method for two linked loci that is based on ASPs. To further utilize affected pairs other than ASPs, Lin and Schaid [8] generalized the two-locus model to ARPs. Because the NARAC (North American Rheumatoid Arthritis Consortium) data contain half siblings (HS) and avuncular pairs (AP) in addition to ASPs, we used the software GEEARP by Lin and Schaid [8] to evaluate whether there are multiple RA-susceptibility genes on chromosome 6.

## Methods

### Data

For the 757 families in the NARAC data, genotypes of 404 single-nucleotide polymorphisms (SNPs) and 27 microsatellites on chromosome 6 were combined for the estimation of IBD. There were 1019 ARPs with genotypes, including 877 full siblings (FS), 45 HS, 13 first cousins (FC), and 84 AP. Only FS, HS, and AP were used in our analyses because each had reasonable sample size. For the Canada-Illumina data, there were 59 ASPs genotyped from 59 families with 404 SNPs on chromosome 6. The French data contained 88 families with 119 ASPs, with 64 microsatellite markers on chromosome 6. We used the first screen of the United Kingdom (UK) data, combining 18 microsatellite markers genotyped on 175 families with 667 SNPs genotyped on 157 families. There were 237 ASPs in the UK data. RA affection status was the only phenotype analyzed.

To avoid biased estimates of IBD from linkage disequilibrium (LD) among high-density SNPs, markers with pair wise *r*^2 ^> 0.5 and all intervening markers were joined into clusters to estimate IBD scores, using the software Merlin (version 1.0.1) [9,10].

### GEEARP analysis

The unknown parameters in the mean function are the locations (τ_1 _and τ_2_) of two susceptibility loci, and genetic effects for each locus (C_1*k *_and C_2*k*_, the effects of genes at τ_1 _and τ_2 _for the *k*^th ^type of ARP). When the two disease susceptibility loci are linked, C_1*k *_and C_2*k *_do not represent the marginal effects of each locus, but rather they incorporate the effect of each other, and thus they change with the recombination fraction between them. When there are several kinds of ARPs, this method involves a large number of parameters (i.e., two genetic effects per ARP type), which can cause considerable variability in parameter estimates, especially when the numbers of the different types of ARPs are small. To reduce the number of parameters, we also fit a constrained model, which constrains the genetic effect of each locus across the different types of ARPs. That is, based on results from Risch [13], the risk ratio λ is a function of genetic effect C, and under no dominance and no epistasis (i.e., genetic relative risks are multiplicative), the genetic effects (C_i*k*_) can be formulated as functions of only one parameter for each locus, λ_*i*_, *i *= 1 or 2. A score test proposed by Schaid et al. [6] tests the homogeneity of risk ratios across different types of ARPs, and has an approximate chi-square distribution. Both unconstrained and constrained models were fit to the pooled data, for both one-locus and two-locus models.

## Results

The estimated gene locations and risk ratios from the one-locus models confirmed linkage at HLA (see Table 1). The unconstrained and constrained models gave similar estimates of location and genetic effects, and there was no evidence of heterogeneity in the genetic risk ratios (*p *= 0.65). Similarly, for the two-locus model (Table 2), the estimates of location and genetic risk ratios were similar between the unconstrained and constrained models, and there was no evidence of heterogeneity in the genetic risk ratios (*p *= 0.77). Because the constrained model provided shorter confidence intervals, this model might provide more precise confidence in gene locations. The two-locus model provides suggestive evidence for two linked regions on chromosome 6, with 95% CIs of 45.6–52.7 (the HLA region) and 54.7–69.6.

**Table 1:** One-locus models for the pooled data: NARAC, Canada-Illumina, France, and the first screen of UK

Type of analysis and ARPs	No. ARP	Estimate (95% CI) for	
		
		*τ *(cM)	*λ*
Unconstrained model			
Full siblings	1292		1.7 (1.4–1.9)
Half siblings	45		2.0 (0.5–3.5)
Avuncular pairs	84		1.2 (0.4–2.1)
Common *τ*		52.4 (49.2–55.7)	
Constrained model	1421	52.4 (49.0–55.8)	1.6 (1.4–1.8)

**Table 2:** Two-locus models for the pooled data: NARAC, Canada-Illumina, France, and the first screen of UK

Type of analysis and ARPs	No. ARP	Estimate (95% CI) for			
		
		*τ*_1 _(cM)	*τ*_2 _(cM)	*λ*_1_	*λ*_2_
Unconstrained model					
Full siblings	1292			1.6 (1.4–1.8)	1.2 (1.0–1.3)
Half siblings	45			1.8 (0.4–3.2)	1.6 (0.6–2.6)
Avuncular pairs	84			1.1 (0.5–1.7)	1.3 (0.3–2.3)
Common *τ*_1_, *τ*_2_		51.3 (48.4–54.3)	79.2 (61.9–96.5)		
Constrained model	1421	49.1 (45.6–52.7)	62.2 (54.7–69.6)	1.5 (1.3–1.7)	1.4 (1.3–1.6)

Despite the evidence of two RA-susceptibility regions on chromosome 6 based on the pooled data, further exploration of the different data sets suggested that not all data were consistent with this model. We fit the two-locus model to each type of ARP from each set of data, and the parameter estimates varied greatly. Note that we could not fit a two-locus model to the French and UK data, because both sets converged to a single-locus model (see Table 3). The large differences in parameter estimates across these strata could result from a number of causes: 1) some strata have small numbers of ARPs; 2) large variability in IBD sharing; 3) hidden biases that influence IBD estimation, such as misspecified allele frequencies; 4) differing information content across the data sets that could influence the fitting of our GEEARP models.

**Table 3:** Models fit to individual data sets

Type of analysis and ARPs	No. ARP	Estimate (95% CI) for			
		
		*τ*_1 _(cM)	*τ*_2 _(cM)	*λ*_1_	*λ*_2_
NARAC					
Full siblings	877	51.8 (48.6–54.9)	84.3 (63.2–105.5)	1.8 (1.4–2.1)	1.2 (1.0–1.4)
Half siblings	45	18.2 (8.8–27.6)	74.2 (58.8–89.6)	2.3 (0.6–4.0)	1.8 (0.6–3.0)
Avuncular pairs	84	8.7 (0.0–17.9)	79.8 (57.8–101.8)	1.6 (0.9–2.4)	1.3 (0.2–2.4)
Canada					
Full siblings	59	44.1 (29.8–58.4)	156.3 (142.8–169.8)	1.5 (0.5–2.5)	1.7 (0.6–2.8)
France					
Full siblings	119	44.9 (33.2–56.7)	NA^a^	1.7 (0.7–2.6)	NA
UK-Screen 1					
Full siblings	237	52.5 (42.8–62.2)	NA	1.5 (1.0–1.9)	NA

^a^The two-locus model converged to the one-locus model

## Discussion and conclusion

We used generalized estimating equations to model the mean IBD sharing of ARPs, such that the mean is as a function of chromosome positions of two susceptibility loci and their genetic effect sizes. Some advantages of our approach are: 1) it is robust in the sense that a genetic model need not be specified, 2) it summarizes information across all types of ARPs (instead of analyses within strata), 3) it estimates gene locations and genetic effects (or transformed to recurrence risk ratios) simultaneously, and 4) it provides 95% CIs of relevant parameters. Although gene location is estimated, the precision can be low because of the nature for coarse mapping in linkage analyses, and if the linkage information content is not high. To identify causative loci, follow-up association analyses in the confidence intervals are necessary. Some cautions about the use of our method based on estimating equations are: 1) it suffers from downward bias in genetic effects (or recurrence risk ratios), which is absent in Kong-Cox LOD scores [11], with a more severe bias toward the null when marker informativeness is low; 2) estimating equations in general can have multiple solutions [12], often found when using different starting values in the iterative algorithm for finding the roots of the function. In our applications, we found two solutions for the NARAC FS subset, the second of which gave a 95% CI for the second linkage region at 110.3–175.8 (instead of the region of 63.2–105.5 listed in Table 3). Although we chose the solution that gave the best fit, in terms of minimum residual sums of squares, it might be safer to follow up both regions. Note that this other solution also agrees somewhat with the subset analysis of the Canadian data set (95% CI 142.8–169.8; see Table 3), giving additional impetus to follow up this region.

Comparing the one- and two-locus models, the one-locus model performed well when the linkage peaks were far apart (close to the situation of unlinked genes) and there is no dominating peak. So, the results of one- and two-locus models were similar in the Canadian data. However, if there is a gene with much larger genetic effect than others, the one-locus model may fail to find other minor linked loci. The results of NARAC-FS and the unconstrained and constrained models showed this. In contrast, the two-locus model allows us to find the second gene in the presence of a much stronger signal. Note, however, that if there is only one gene in a region, the two-locus model can be over-parameterized and lose statistical efficiency. With these considerations, we suggest fitting both one- and two-locus models to evaluate linkage of multiple genes in a region, and our GEEARP software provides both of these analyses.

## List of Abbreviations

AP: avuncular pair

ARP: affected relative pair

ASP: affected sib pair CIs: confidence intervals

FC: full cousin

FS: full sibling

GAW15: Genetic Analysis Workshop 15

GEE: generalized estimating equation

HS: half sibling

IBD: identical by descent

LD: linkage disequilibrium

NARAC: The North American Rheumatoid Arthritis Consortium

RA: rheumatoid arthritis

SNP: single-nucleotide polymorphism

## Competing interests

The author(s) declare that they have no competing interests.
